# Free Silicone With Giant Cell Reaction Can Enhance on Breast MRI

**DOI:** 10.7759/cureus.29365

**Published:** 2022-09-20

**Authors:** Brittany L Miles, Quan D Nguyen

**Affiliations:** 1 Medical Education, University of Texas Medical Branch, Galveston, USA; 2 Radiology, Baylor College of Medicine, Houston, USA

**Keywords:** implant rupture, gadolinium enhancement mri, bi-rads, mri - magnetic resonance imaging, silicone breast prostheses

## Abstract

Background

Breast augmentation with silicone implants is commonplace, and such implants have a risk of rupture which increases over time. Most implant ruptures are asymptomatic, and magnetic resonance imaging (MRI) is a recommended imaging modality for surveillance to detect these events. If a silicone leak enhances on MRI, it is currently categorized according to the Breast Imaging Reporting and Data System (BI-RADS) as category 4, which results in a recommendation for biopsy even when free silicone leakage is the most likely diagnosis. In this article, we present a case series that illustrates this issue with the BI-RADS system and propose an algorithmic approach that may allow some patients to be placed into BI-RADS category 3 and avoid biopsy.

Methodology

Eight cases of silicone breast implant rupture were identified at the University of Texas Medical Branch at Galveston over a five-year period. Two cases were excluded because MRI was not performed. The remaining six cases were evaluated for history and physical findings as well as mammogram, ultrasound, and MRI. All identified cases had been categorized as BI-RADS 4 and underwent biopsy.

Results

The six cases in this series exhibited pre-biopsy radiographic findings that were most consistent with silicone implant rupture. The ruptures were proven by biopsy, and no evidence of malignancy was identified in any of the patients.

Conclusions

Free silicone from breast implant rupture can present with enhancement on MRI. The two main categories of breast MRI enhancement, namely, mass and non-mass, include malignancies in their differential diagnoses and result in a BI-RADS category 4 designation. By correlating the findings with other imaging modalities, some of these patients can be classified as BI-RADS category 3 and biopsy can safely be avoided.

## Introduction

Breast augmentation is a common procedure, both worldwide and in the United States. In 2019, the American Society of Plastic Surgeons reported more than 287,000 breast augmentations, and 137,808 breast reconstruction procedures were performed. Overall, 84% of the breast implant procedures were performed using silicone implants, while 16% were saline [1]. The silicone inside implants manufactured prior to 1992 consists of a liquid silicone formulation that imparts a relatively high risk of leakage external to the implant in the event of implant rupture [2]. Silicone implants used in the United States since 2006 contain a silicone gel and are considered less likely to leak free silicone in comparison to the older generation of implants. Many women who experienced silicone leakage from the older generation of implants reported symptoms consistent with connective tissue disorders, including fatigue, joint pain, muscle pain, and weakness despite having none of those symptoms prior to augmentation [3]. A meta-analysis of studies investigating a possible connection between silicone implants and connective tissue disease found no such correlation [4]. The determination of whether silicone leakage is intracapsular or extracapsular depends on whether it has crossed the fibrous capsule that had developed around the implant prior to rupture [5]. The rupture of a silicone gel implant is most likely to be detected by magnetic resonance imaging (MRI), which is recommended to be performed on a routine basis for monitoring the development of such an event [6].

## Materials and methods

Inclusion and exclusion criteria

Cases of silicone implant rupture were identified by a breast radiologist in the Department of Radiology at the University of Texas Medical Branch at Galveston (UTMB) from 2016 through 2021. The inclusion criteria for this case series consisted of a diagnosis of silicone implant rupture. The exclusion criteria included the absence of magnetic resonance (MR) images for review. Mammography, ultrasound imaging, and biopsy were not required for inclusion but were present in all cases analyzed. Eight cases were identified for evaluation, but two were excluded due to an absence of MR images.

Study design

The purpose of this case series is to evaluate whether silicone implant rupture can be diagnosed based on imaging characteristics, allowing some patients with this condition to avoid undergoing biopsy. It was noticed at our institution that free silicone with giant cell reaction can enhance on MRI and that current Breast Imaging Reporting and Data System (BI-RADS) guidance is for such enhancement to be considered “suspicious” and for biopsy to be recommended despite strong pre-biopsy suspicion for silicone rupture as the most likely diagnosis.

Data collection

The history and physical examination information for each case were reviewed to determine the circumstances that resulted in imaging studies being ordered. Physical examination information was also evaluated to determine if patients were symptomatic or asymptomatic at presentation. In each case, the imaging studies were reviewed and relevant images were obtained for inclusion in this series. Images for each patient were aggregated using Adobe Photoshop, with letters and arrows added for picture identification and designation of areas of interest, respectively.

## Results

Case one

**Figure 1:**
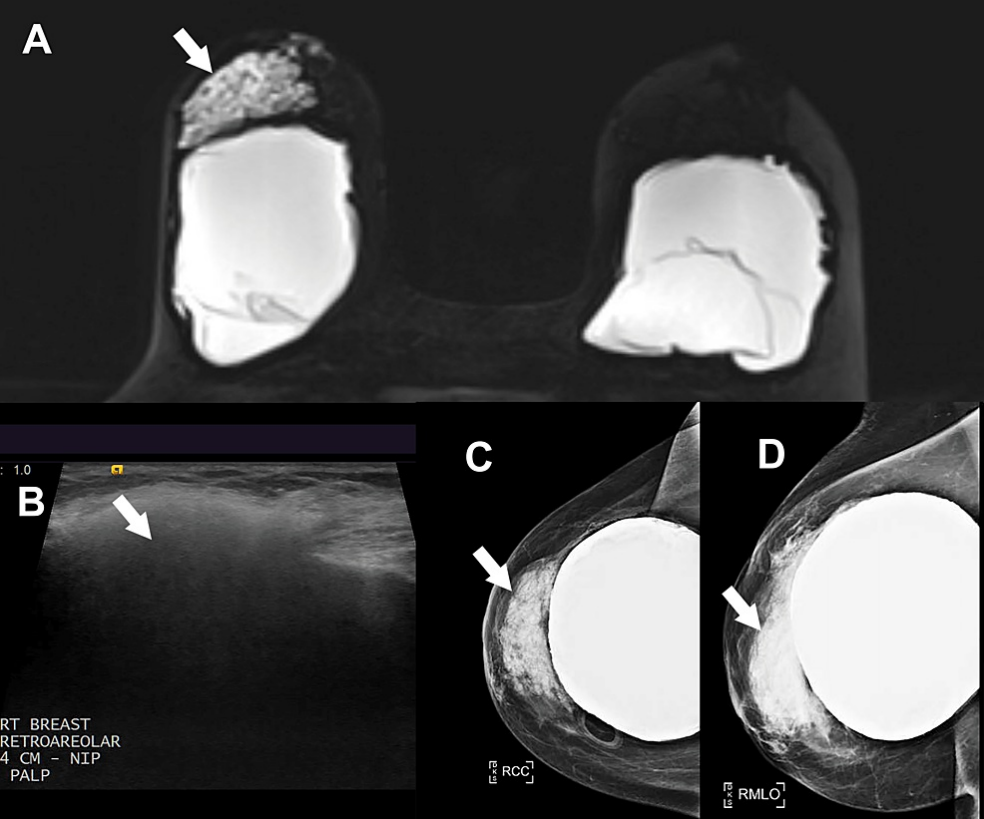
(A) Heterogeneous non-mass magnetic resonance imaging enhancement measuring 50 × 86 × 46 mm. (B) Ultrasound demonstrates heterogeneous echogenicity with dispersion of the ultrasound beam (“snowstorm appearance”). (C) Craniocaudal and (D) mediolateral oblique view mammogram images showing high density in the central right breast without any suspicious masses or calcifications.

A 66-year-old woman with a history of breast implant surgery 32 years prior presented for evaluation of a known right-sided implant rupture. One year prior to the presentation, she self-discovered a mass in the right breast and was evaluated with a mammogram and ultrasound, and the diagnosis of implant rupture was made (Figure 1).

These findings were classified as BI-RADS category 4A (suspicious for malignancy). Because the area of non-mass enhancement was not visible on ultrasound, an MRI-guided biopsy was recommended. A core needle biopsy of the 10 o’clock lesion revealed a foreign body giant cell reaction and chronic inflammation. No atypical hyperplasia, in-situ carcinoma, or invasive carcinoma was identified. Because her skin envelopes were thin and she was an active smoker, she was at risk for vascular compromise, and surgery was not offered.

Case two

**Figure 2:**
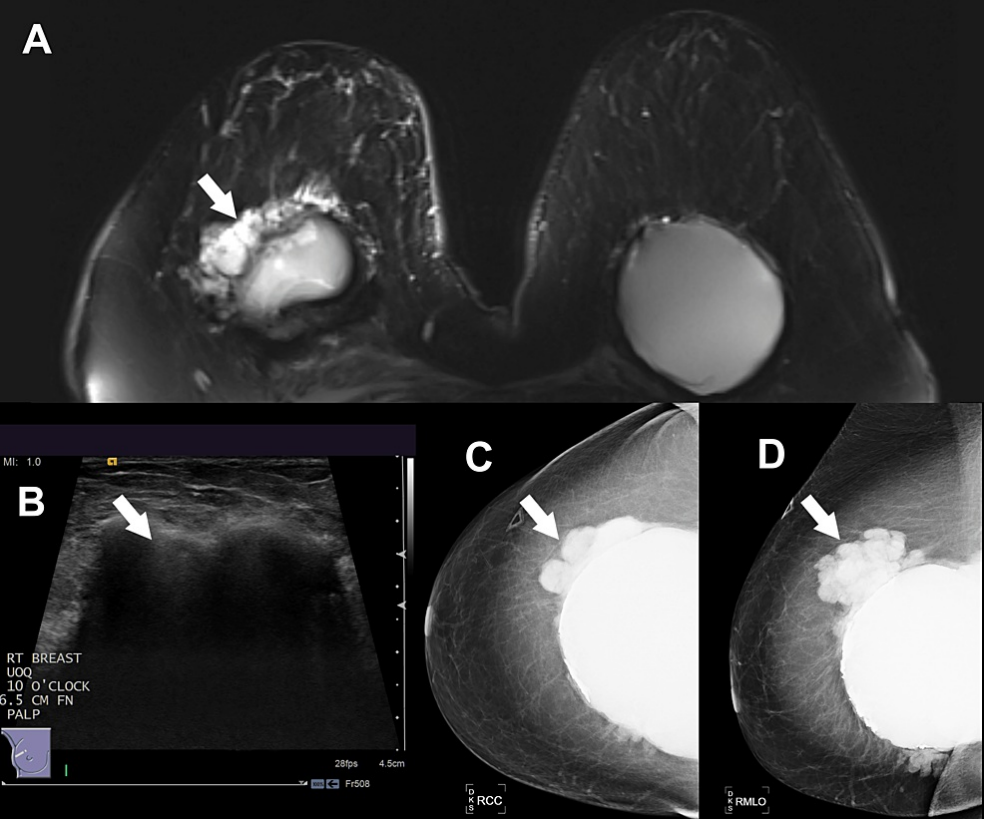
(A) Magnetic resonance imaging showing heterogeneous, lobular pericapsular enhancement at the 10 o’clock position 6.5 cm from the nipple, measuring 45 × 70 × 50 mm. (B) Ultrasound demonstrating a snowstorm appearance in the same location. (C) Craniocaudal and (D) mediolateral oblique mammography views showing extracapsular silicone rupture and medial herniation.

A 70-year-old woman was found on routine breast screening to have a suspicious palpable lesion on the right breast (Figure 2).

Mammogram, ultrasound, and MRI were consistent with extracapsular silicone from implant rupture, without any radiographic concern for malignancy. The abnormal enhancement was reported as BI-RADS category 4, and a biopsy was recommended. The pathology revealed chronic inflammation, fat necrosis, and foreign body giant cell reaction consistent with silicone granuloma. No malignancy was identified.

Case three

**Figure 3:**
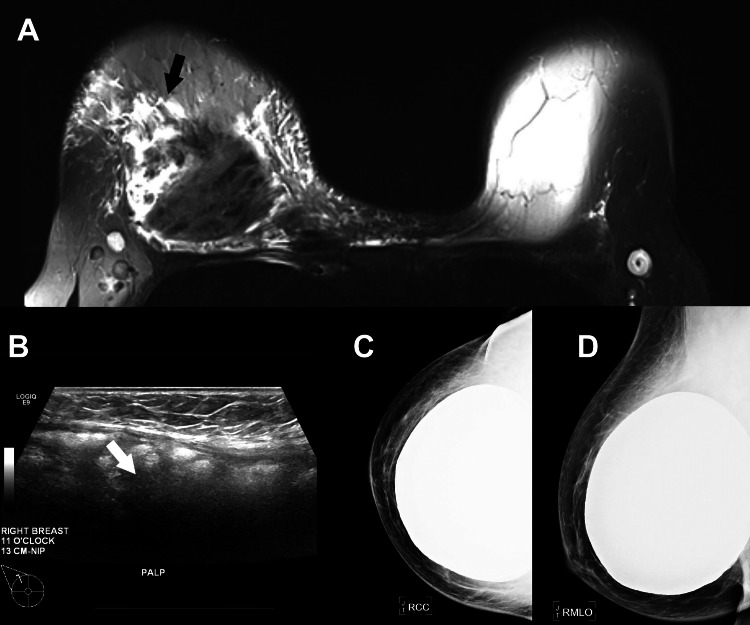
(A) Magnetic resonance imaging shows prepectoral silicone implant with intracapsular rupture, with free silicone and associated non-mass enhancement likely representing granulomatous reaction. (B) Ultrasound showing snowstorm appearance consistent with free silicone. (C) Craniocaudal and (D) mediolateral oblique mammogram views did not show free silicone (likely obscured by the overlying implant).

A 71-year-old woman presented to her primary provider with a complaint of a self-discovered right breast mass. She was referred for breast imaging (Figure 3).

The MRI and ultrasound imaging were consistent with free silicone from implant rupture, although free silicone was not visible on the mammogram, likely due to obscuration from the overlying implant. The imaging findings were reported as suspicious (BI-RADS Category 4A), and a biopsy was recommended. The pathology report indicated granulomatous inflammation, silicone material, and foreign body-type giant cell reaction with no evidence of malignancy.

Case four

**Figure 4:**
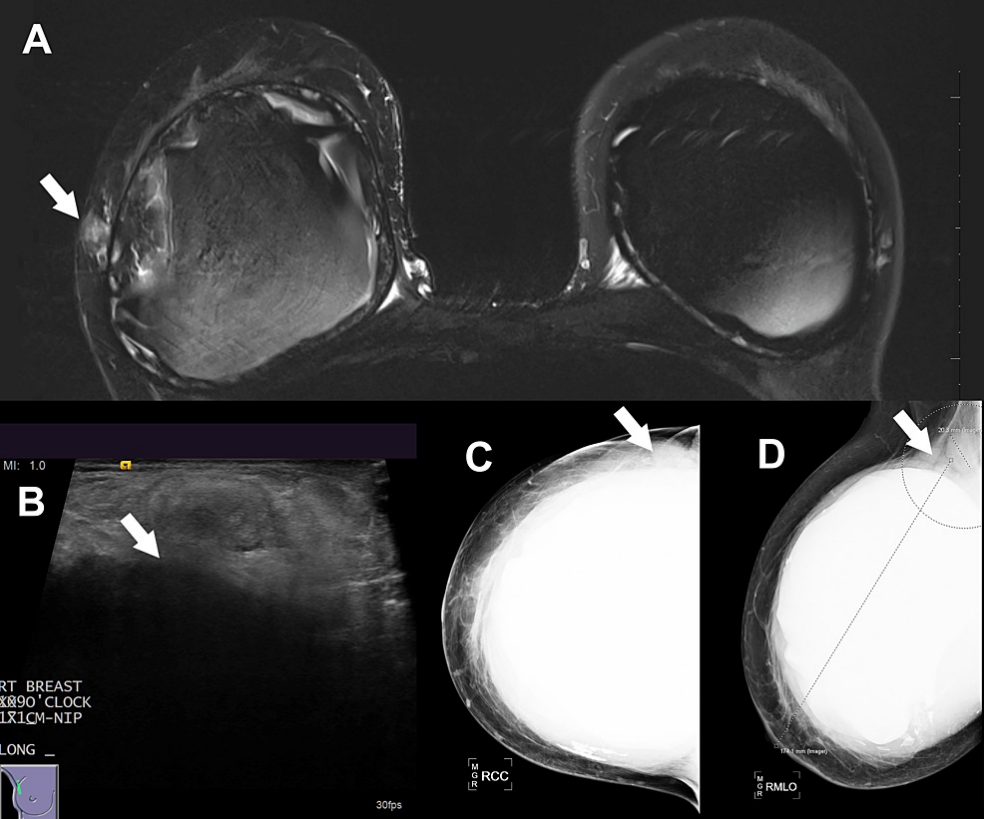
(A) Magnetic resonance imaging with enhancement at the 9 o’clock position of the right breast. (B) Ultrasound with a snowstorm appearance consistent with silicone. (C) Craniocaudal and (D) mediolateral oblique mammogram views showing possible extracapsular silicone and a dominant right axillary lymph node.

A 65-year-old woman was found to have complex peri-implant fluid collections and was referred for additional breast imaging (Figure 4).

An area of MRI enhancement was seen in the lateral right breast, and correlation with the mammogram and ultrasound images was consistent with free silicone from implant rupture. The abnormalities were reported as BI-RADS category 4, with the recommendation to consider biopsy. A prominent right axillary lymph node which was identified on mammography was biopsied and found to contain lipogranulomas, refractile material, and benign fibroadipose tissue with no evidence of malignancy.

Case five

**Figure 5:**
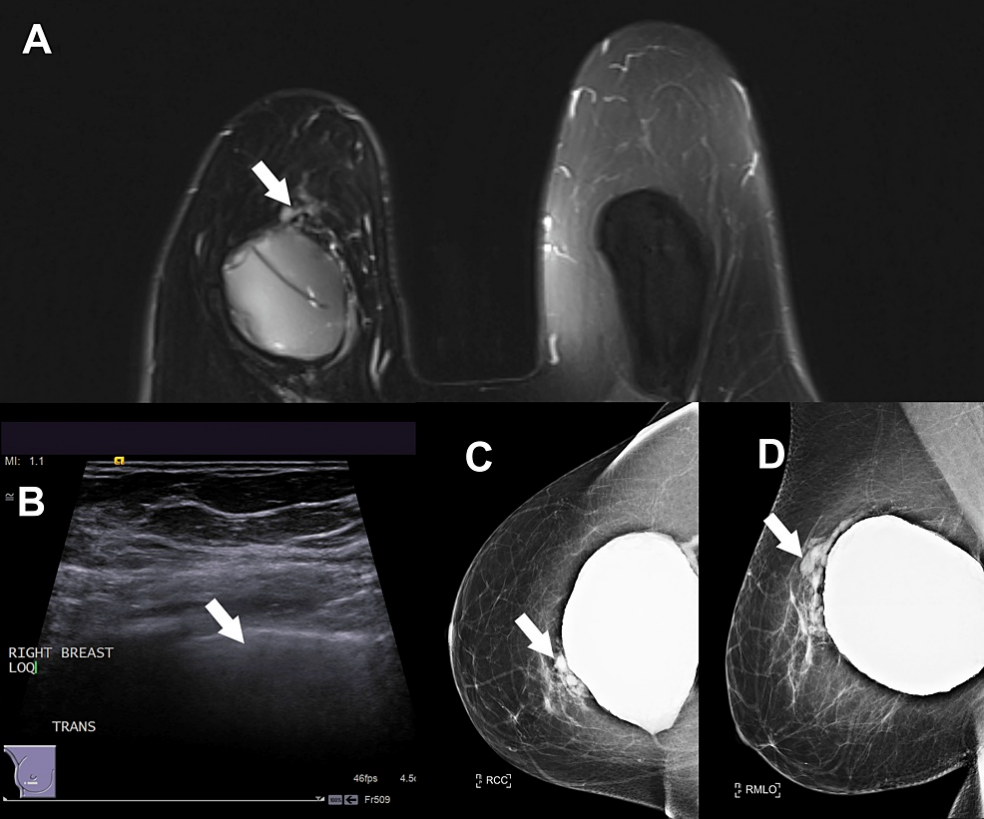
(A) Magnetic resonance imaging shows intracapsular and extracapsular rupture. (B) Ultrasound of the affected area revealed dispersion consistent with the presence of extracapsular silicone. (C) Craniocaudal and (D) mediolateral oblique mammogram images are consistent with implant rupture and the presence of extracapsular silicone.

A 73-year-old female who was previously seen by plastic surgery for a ruptured silicone implant requested a second opinion. On physical examination, she was found to have a pea-sized nodule along the edge of the implant, and additional imaging was performed (Figure 5).

Imaging was consistent with bilateral silicone implant rupture, and the patient requested surgical removal of both implants. Foreign body giant cell reactions containing silicone were present bilaterally.

Case six

**Figure 6:**
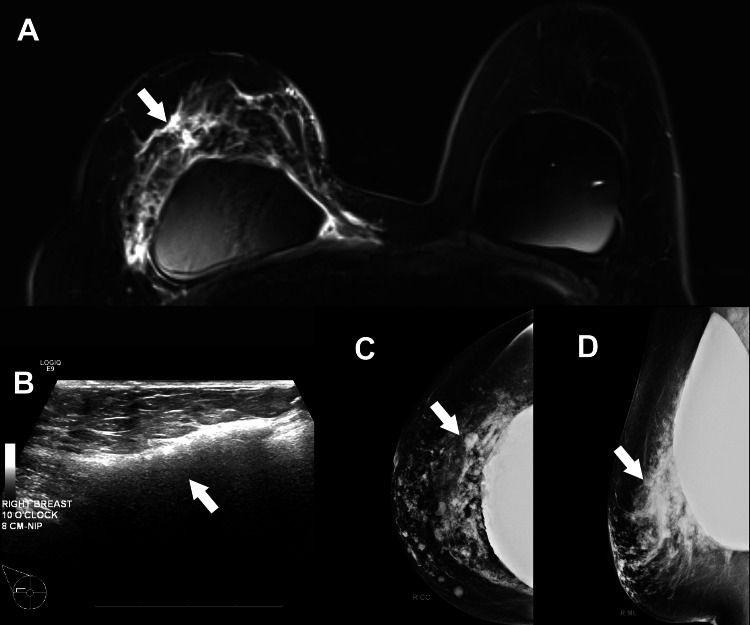
(A) Right breast magnetic resonance imaging showing extensive extracapsular non-mass enhancement. (B) Ultrasound showed multiple areas of snowstorm-type dispersion consistent with free silicone. (C) Craniocaudal and (D) mediolateral oblique mammogram images showing numerous areas consistent with silicone granulomas.

A 57-year-old female presented with a two-month history of pain, redness, and warmth of the right breast and was concerned about possible implant rupture. A mammogram and ultrasound at an outside facility confirmed the implant rupture, but the patient desired evaluation at a larger institution, and repeat imaging was performed (Figure 6).

The imaging was reported as BI-RADS category 4A, recommending consideration of biopsy but with low suspicion for malignancy. Core needle biopsies were performed, indicating refractile foreign material with giant cell reaction and chronic inflammation, consistent with silicone granulomas.

## Discussion

Recent studies have shown that the newer generation of silicone gel implants degrade over time, with changes in color, clarity, and silicone cohesion [7]. These changes gradually increase the risk of silicone release following a rupture event, eventually resulting in no statistical difference in silicone leakage rates between the newer gel and older liquid silicone implants [7,8]. This may be an early indication that silicone leakage from breast implant rupture may not just be a problem of the past but an ongoing issue into the future.

MRI is regarded as a highly accurate modality for the evaluation of breast implant rupture, with a sensitivity of 80-90% and specificity of 90-97% [9]. On MRI, silicone does not enhance well on fast spin-echo T2-weighted imaging. Instead, water-suppressed inversion-recovery T2-weighted images are preferred for better visualization of extracapsular silicone [10]. With fat and water-suppression techniques, MRI can provide optimal visualization of free silicone and should permit the differentiation of free silicone from a breast neoplasm [11].

It has been previously reported that the level of enhancement on water-suppressed MRI of a silicone implant leak is proportional to the amount of silicone present in tissue and that the degree of enhancement is expected to be less than or equal to that of the silicone remaining within the implant [12]. It is reasonable to expect that larger granulomatous reactions may occur in the setting of a greater amount of free silicone release, which could be either time-dependent or a reflection of the magnitude of trauma that caused the implant rupture to occur. Because implant ruptures are usually asymptomatic, it is often difficult to know how long a rupture has been present [2].

The American Journal of Roentgenology’s primer on breast MRI delineates three categories of enhancement: foci, mass, and non-mass [13]. The current approach to enhancement on breast MRI is to classify it as BI-RADS category 4, suspicious for malignancy, and biopsy is frequently performed [14]. This is because the mass and non-mass enhancement categories include malignancy in their differential diagnoses and do not include the diagnosis of free silicone with giant cell reaction [15,16]. The mass enhancement differential includes ductal carcinoma in situ, invasive ductal carcinoma, and infiltrating lobular carcinoma. The non-mass enhancement category includes the same three potential diagnoses, as well as benign entities which can be difficult or impossible to distinguish from malignancy radiographically.

The BI-RADS classification is used to report the risk of malignancy on breast imaging and guide as to whether abnormal findings should be evaluated with biopsy [14]. In mammography, BI-RADS category 4 is divided into three subgroups based on the likelihood of malignancy: 4A (>2% to ≤10%), 4B (>10% to ≤50%), and 4C (>50% to <95%). Category 4 is not subdivided for breast MRI due to a paucity of available data [17]. Nevertheless, because a biopsy of a breast lesion is recommended if the risk of cancer is greater than 2%, any enhancing category 4 lesion will prompt a recommendation for biopsy.

By recognizing that free silicone and giant cell reactions can enhance on MRI, a useful algorithm could be to compare the area of MRI enhancement to mammogram and ultrasound images to confirm the presence of silicone and report that the enhancement is probably benign (BI-RADS category 3 instead of 4). Most BI-RADS 3 abnormalities are not recommended to undergo biopsy, and short-term follow-up with imaging in six months is recommended. If the initial comparison yields uncertainty, then water-suppressed inversion-recovery T2-weighted MRI imaging can be performed for additional reassurance that silicone leakage is the underlying cause of the enhancement. Biopsy could then be safely avoided in some of these patients. This systematic approach in which radiographic evaluation may obviate the need for some biopsies has the potential to save the time, discomfort, and expense associated with those procedures.

The primary challenge is that the BI-RADS 3 category has been described as the most difficult assessment category to use and has a high level of interobserver variability [18]. For breast MRI, criteria for the use of BI-RADS category 3 are also not well-established, and the data to support the use of BI-RADS 3 for non-mass MRI enhancement are very limited [18]. Low risk of cancer has been associated with non-mass enhancement that possesses homogeneous internal enhancement and a regional distribution, and the use of BI-RADS category 3 may therefore be appropriate for patients with free silicone release from implant rupture.

## Conclusions

Breast implant rupture is often asymptomatic, and MRI surveillance for monitoring silicone implant integrity is not commonly performed. Extracapsular silicone leakage may induce a giant cell foreign body reaction which can enhance on MRI, especially on water-suppressed inversion-recovery T2-weighted images. The degree of enhancement correlates to the density of silicone present in the tissues and can often indicate the presence of a foreign body giant cell reaction.

Silicone implant rupture is a unique problem that may benefit from being treated differently from other types of breast MRI enhancement. Correlation with mammogram, ultrasound, or water-suppressed MRI may confirm that the enhancement is due to free silicone release or a giant cell foreign body response to implant rupture, allowing some patients to avoid an unnecessary biopsy procedure.

## References

[1] Plastic Surgery Statistics (2022). American Society of Plastic Surgeons. Plastic surgery statistics. https://www.plasticsurgery.org/news/plastic-surgery-statistics.

[2] Hillard C, Fowler JD, Barta R, Cunningham B (2017). Silicone breast implant rupture: a review. Gland Surg.

[3] American Society for Aesthetic (2022). American Society for Aesthetic Plastic Surgery. Breast implant illness - frequently asked questions/talking points. August.

[4] Lipworth L, Tarone RE, McLaughlin JK (2004). Silicone breast implants and connective tissue disease: an updated review of the epidemiologic evidence. Ann Plast Surg.

[5] Swezey E, Shikhman R, Moufarrege R (2022). Breast Implant Rupture. https://www.ncbi.nlm.nih.gov/books/NBK459308/.

[6] Tanne JH (2006). FDA approves silicone breast implants 14 years after their withdrawal. BMJ.

[7] Bodin F, Jung C, Dieval F, Chakfe N, Wisniewski S, Bruant Rodier C, Heim F (2015). Aging of retrieved gel breast implants: a comparison between two product generations. J Mech Behav Biomed Mater.

[8] Dijkman HB, Slaats I, Bult P (2021). Assessment of silicone particle migration among women undergoing removal or revision of silicone breast implants in the Netherlands. JAMA Netw Open.

[9] Juanpere S, Perez E, Huc O, Motos N, Pont J, Pedraza S (2011). Imaging of breast implants-a pictorial review. Insights Imaging.

[10] Berg WA, Nguyen TK, Middleton MS, Soo MS, Pennello G, Brown SL (2002). MR imaging of extracapsular silicone from breast implants: diagnostic pitfalls. AJR Am J Roentgenol.

[11] (2022). Learning Radiology. Free silicone injections. https://learningradiology.com/notes/mammonotes/siliconeinjections.htm.

[12] Middleton MS (2014). MR evaluation of breast implants. Radiol Clin North Am.

[13] Erguvan-Dogan B, Whitman GJ, Kushwaha AC, Phelps MJ, Dempsey PJ (2006). BI-RADS-MRI: a primer. AJR Am J Roentgenol.

[14] (2022). Breast Imaging Reporting & Data System. https://www.acr.org/Clinical-Resources/Reporting-and-Data-Systems/Bi-Rads.

[15] Chadashvili T, Ghosh E, Fein-Zachary V, Mehta TS, Venkataraman S, Dialani V, Slanetz PJ (2015). Nonmass enhancement on breast MRI: review of patterns with radiologic-pathologic correlation and discussion of management. AJR Am J Roentgenol.

[16] Glassman L, Hazewinkel M (2022). Radiology Assistant. MRI of the breast. https://radiologyassistant.nl/breast/mri/mri-of-the-breast.

[17] Strigel RM, Burnside ES, Elezaby M, Fowler AM, Kelcz F, Salkowski LR, DeMartini WB (2017). Utility of BI-RADS assessment category 4 subdivisions for screening breast MRI. AJR Am J Roentgenol.

[18] Lee KA, Talati N, Oudsema R, Steinberger S, Margolies LR (2018). BI-RADS 3: current and future use of probably benign. Curr Radiol Rep.

